# Phenolic Acids Released in Maize Rhizosphere During Maize-Soybean Intercropping Inhibit *Phytophthora* Blight of Soybean

**DOI:** 10.3389/fpls.2020.00886

**Published:** 2020-07-28

**Authors:** He Zhang, Yuxin Yang, Xinyue Mei, Ying Li, Jiaqing Wu, Yiwen Li, Huiling Wang, Huichuan Huang, Min Yang, Xiahong He, Shusheng Zhu, Yixiang Liu

**Affiliations:** ^1^State Key Laboratory for Conservation and Utilization of Bio-Resources in Yunnan, Yunnan Agricultural University, Kunming, China; ^2^Key Laboratory for Agro-Biodiversity and Pest Control of Ministry of Education, College of Plant Protection, Yunnan Agricultural University, Kunming, China; ^3^China France Plantomix Joint Laboratory, Yunnan Agricultural University, Kunming, China

**Keywords:** intercropping, *Phytophthora sojae*, phenolic acids, interference, infection behavior

## Abstract

Interspecies interactions play a key role in soil-borne disease suppression in intercropping systems. However, there are limited data on the underlying mechanisms of soil-borne *Phytophthora* disease suppression. Here, a field experiment confirmed the effects of maize and soybean intercropping on *Phytophthora* blight of soybean caused by *Phytophthora sojae*. Experimentally, the roots and root exudates of maize were found to attract *P. sojae* zoospores and inhibit their motility and the germination of cystospores. Furthermore, five phenolic acids (*p*-coumaric acid, cinnamic acid, *p*-hydroxybenzoic acid, vanillic acid, and ferulic acid) that were consistently identified in the root exudates and rhizosphere soil of maize were found to interfere with the infection behavior of *P. sojae*. Among them, cinnamic acid was associated with significant chemotaxis in zoospores, and *p*-coumaric acid and cinnamic acid showed strong antimicrobial activity against *P. sojae*. However, in the rhizosphere soil of soybean, only *p*-hydroxybenzoic acid, low concentrations of vanillic acid, and ferulic acid were identified. Importantly, the coexistence of five phenolic acids in the maize rhizosphere compared with three phenolic acids in the soybean rhizosphere showed strong synergistic antimicrobial activity against the infection behavior of *P. sojae*. In summary, the types and concentrations of phenolic acids in maize and soybean rhizosphere soils were found to be crucial factors for *Phytophthora* disease suppression in this intercropping system.

## Introduction

Intercropping, or the practice of growing two or more crops in the same field, is widely used in Asia, Latin America, and Africa, providing as much as 15–20% of the global food supply (Machado, 2009; Lithourgidis et al., 2011). Intercropping can increase yield stability by increasing species diversity of farmland ecosystems but also effectively alleviate the prevalence of and damage by pests and diseases (Ratnadass et al., 2012; Lv et al., 2018). Many previous studies have shown that intercropping could control the occurrence of airborne crop diseases by forming a physical barrier, diluting pathogens, and improving field microclimates while effectively inhibiting soil-borne diseases through root interactions (Zhu et al., 2000, 2005; Yang et al., 2014). For example, maize/pepper, tomato/chives, watermelon/rice, and wheat/broad bean intercropping can reduce the damage from pepper *Phytophthora* blight, tomato bacterial wilt, watermelon Fusarium wilt, and wheat take-all, respectively (Yu, 1999; Hao et al., 2010; Wang et al., 2016).

Interspecies interactions that occur during intercropping can lead to the suppression of soilborne diseases. Plant roots interact with many soil-inhabiting microbes that can colonize them and provide plants with key functions for plant longevity and fitness (Pascale et al., 2020). Root-derived exudates act as vital food sources or signals for microbes, and they not only support microbial proliferation in the rhizosphere but are also responsible for the formation of distinct microbial assemblages between soil and the rhizosphere (Berendsen et al., 2012; Lombardi et al., 2018; Olanrewaju et al., 2019). For example, benzoxazinoids and triterpenes from plant root exudates could optimize the microbial community in the plant rhizosphere, which helped plants to resist pathogens (Hu et al., 2018; Huang et al., 2019). Coumarins can attract *Pseudomonas* into the plant rhizosphere and then also reshape the composition of the microbiome community around the roots (Stringlis et al., 2019; Pascale et al., 2020). Intercropped maize has been found to cause a twofold increase in flavonoid exudations and increased soybean nodulation by *Rhizobium* (Li B. et al., 2016).

Apart from supporting beneficial associations with soil-inhabiting microbes, non-host plant roots could also interfere with the infections caused by pathogenic microorganisms in intercropping systems (Hao et al., 2010; Lv et al., 2018; Rolfe et al., 2019). Previous studies have found that maize roots could attract *Phytophthora capsici* zoospores, simultaneously inhibiting zoospore swimming and cystospore generation, which helps peppers to resist *Phytophthora* blight (Yang et al., 2014). This phenomenon has also been found in the interaction between other non-host plant species and *Phytophthora* (Fang et al., 2016; Jiang et al., 2017), and it may be an important factor that is involved in soil-borne *Phytophthora* pathogen suppression in intercropping systems. Thus, we infer that the key compounds of non-host root exudates may mediate the interactions of pathogen infection processes, and their underlying mechanisms remain to be further studied.

Fungal and oomycete pathogens have been known to orient hyphal growth and zoospore swimming toward chemical stimuli from the host plant (Turra et al., 2015). Additionally, root exudates also act as chemoattractants to recruit beneficial microorganisms (Lombardi et al., 2018). For example, isoflavones released by soybean roots attract the nodulating symbiont *Bradyrhizobium japonicum* as well as the pathogen *Phytophthora sojae*, which causes *Phytophthora* blight in soybean (Morris et al., 1998). The chemotaxis of pathogenic and beneficial microbes to plant roots could be used in intercropping systems to help the non-host plant to inhibit pathogens. Previous studies have found that *Phytophthora* zoospores display chemotaxis toward the roots of many non-host plants, including chives, rape, and maize, indicating that this attraction phenomenon occurs widely and may be caused by some common compounds in root exudates such as sugars, amino acids, phenolic acids, etc. (Yang et al., 2014; Fang et al., 2016; Jiang et al., 2017). Phenolic acids, which are defined chemically as carboxylic acids derived from either benzoic or cinnamic acid skeletons and can be divided into hydroxybenzoic acid and hydroxycinnamic acid (Stalikas, 2007), are aromatic secondary plant metabolites that are widely distributed in plant root exudates (Herrmann, 1989; Shahidi and Ambigaipalan, 2015). A number of previous studies have found that phenolic acids can promote the growth of plant pathogens and play an important role in the continuous soil sickness of many plants (Ye et al., 2004; Zhou et al., 2012). However, some studies also reported the antifungal activity of phenolic acids *in vitro* (Zhang et al., 2006; Wu et al., 2009). These different effects of phenolic acids on pathogens may result from the chemical structure of phenolic acids associated with various plants. Hence, we hypothesize that the special type or concentration of phenolic acids may be an important factor during the suppression of the *Phytophthora* infection process.

In the present work, we used the “corn/soybean-*Phytophthora sojae*” model as the research object to conduct the following study. First, we performed a field study to understand the inhibition of *Phytophthora* blight in soybean by maize/soybean intercropping. We then observed the interaction between roots/root exudates and pathogens and identified the phenolic acids in the rhizosphere soils of maize and soybean. Finally, we tested the antimicrobial activity of phenolic acid compounds on the infection behavior according to their concentrations in the rhizosphere soils of maize and soybean revealing the underlying mechanism of infection behavior suppression in *P. sojae* by non-host plants.

## Materials and Methods

### Plant and Pathogen Materials

The maize (B73) and soybean (Williams) used in the present study were provided by the State Key Laboratory for Conservation and Utilization of Bio-Resources at Yunnan Agricultural University. *P. sojae* (P6497) was provided by the Seed Pathology and Pharmacology Laboratory of China Agricultural University. A zoospore suspension was obtained using a previously described trapping method with a few modifications (Lan et al., 2007). *P. sojae* was grown on V8 medium in a 25°C incubator under a 12-h light-dark cycle for 7 days (Hua et al., 2008). Eight samples were taken from the colony edges, seeded into a 250 mL culture flask containing 10% liquid V8 medium, and shaken in the dark at 25°C and 140 rpm for 48 h. All the cultures were transferred to new culture dishes and washed with sterile water four times. After the washing, the hyphae were added to 15 ml of soil extract and cultured at 25°C in the dark for 12–15 h, and then the zoospores were released. A zoospore suspension (10^6^ mL^–1^) was prepared after the zoospores were filtered through gauze and counted using a hemocytometer.

### Field Experiment

**FIGURE 1:**
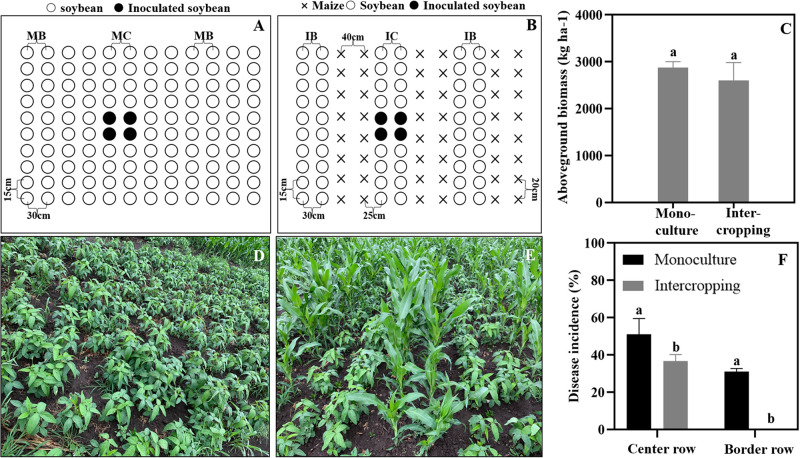
Maize and soybean intercropping patterns in the field and their effects on soybean *Phytophthora* blight. **(A,D)** Soybean monoculture; **(B,E)** Maize and soybean intercropping. **(C)** Aboveground biomass of soybean in monoculture and intercropping schemes. **(F)** Disease incidence of soybean *Phytophthora* blight in monoculture and intercropping systems. IB and MB indicate the border lines (indicator line) in the intercropping and monoculture fields. IC and MC indicate the center lines (inoculation line) in intercropping and monoculture fields. Significant differences are based on ANOVA tests (*P* < 0.05). The error bars indicate the standard errors of the means (*n* = 3).

A field trial was conducted in 2019 at the Harbin Experimental Station of Heilongjiang Academy of Agricultural Sciences (45°50′N, 126°51′E), Heilongjiang Province, China. To determine the effect of the soybean and maize intercropping system on soybean *Phytophthora* blight suppression, we conducted field experiments using a single factor randomized block design with three replicates. The treatments are shown in Figure 1, and they include soybean monoculture and maize/soybean intercropping. The area of the individual plots was 3.6 × 5.6 m^2^. A maize/soybean intercrop was planted in a west-east row orientation in alternating 120-cm-wide strips, which included a 60 cm maize strip (two rows of maize with 40 cm inter-row spacing and 20 cm intra-row distance) and a soybean strip (two rows of soybean with 30 cm of inter-row spacing and 15 cm of intra-row distance). The wide gap between the maize and soybean strips measured 25 cm. We then used the same inter- and intra-row spacing (30 and 15 cm) for soybean in the monoculture. Each treatment was replicated three times. All the plots were located in the same field and arranged using a randomized block design. When the soybean plants had grown to the three-leaf stage, the cotyledon hypocotyl method described by Zuo (2004) was used to inoculate the soybean in the field according to Figure 1. In brief, the soybean plants were cut with a blade at 0.5 cm below the cotyledon, and then the inoculum was injected into the wound. The incidence rates of soybean *Phytophthora* disease in the center and border rows were surveyed to show the ability of the zoospores to spread in and across rows. The incidence was calculated by using the following formula:

Disease incidence rate of soybean in the center row = Number of infected plants in center row/Total number of investigated plants in center row × 100%.

Disease incidence rate of soybean border row = Number of infected plants in border row/Total number of investigated plants in border row × 100%.

Simultaneously, all the aboveground samples were sun-dried and weighed to calculate the aboveground soybean biomass.

### Interaction Assay Between Maize Roots and *P. sojae*

A special apparatus was used to monitor the interaction between the spores of *P. sojae* and the maize roots (Yang et al., 2014). In brief, a U-shaped chamber was formed by placing a bent capillary tube on a glass slide and covering it with a coverslip. The maize roots, which were approximately 2 cm long, were excised with a sterile razor blade. The root cap side of the maize roots was inserted into a zoospore suspension (10^6^mL^–1^) in the chamber. The behavior of the zoospores in the rhizosphere was recorded every minute for a period of 5 min by taking five photographs of the rhizosphere along the root cap and elongation zone under a light microscope (Leica DM2000, Germany) and adjusting the magnification (40 times or 100 times) according to the test requirement for photo collection. A capillary tube was inserted into a chamber containing the same zoospore suspension concentration as the control. The numbers of total cystospores and germinating cystospores in the rhizosphere were counted. Then, the inhibition ratio of the swimming zoospores and the cystospores germination were calculated using the follow formulas: Inhibition ratio of zoospores swimming = (Number of zoospores in the control – Number of zoospores in the treatment)/Number of zoospores in the control × 100%; and Inhibition ratio of cystospores germination = Number of germinated cystospores in the control – Number of germinated cystospores in the treatment)/Number of germinated cystospores in the control × 100%, respectively. The chemotactic ratio (CR) was calculated as “the numbers of the zoospores and cystospores on the test root” divided by “the numbers of the zoospores and cystospores in the control” (Halsall, 1976). A CR > 1 indicates positive chemotactic activity. Each interaction assay was replicated three times.

### Antimicrobial Activity of Maize Root Exudates and Phenolic Acids Against *P. sojae*

#### Collection of Maize Root Exudates

Maize plants were cultured using a previously described method (Yang et al., 2014), and root exudates were collected by water culture method. The maize seeds were surface sterilized with 3% sodium hypochlorite for 10 min and sown in black plastic pots with 40% humus soil and 60% field soil (poor soil outside the planting area). The 60% poor field soil were used to ensure the similarity with field soil where pathogen suppression was observed. And the 40% humus was used to supplement nutrient to avoid the effect of nutritional competition on root exudates. One maize seed was sown in each pot and irrigated with water. When the maize reached the three-leaf stage, the maize plant from each pot was removed and washed, and the maize roots were immersed in 200 mL of distilled water for 4 h to collect the root exudates. The collected liquids were filtered and extracted twice with ethyl acetate and concentrated under reduced pressure (Rotavapor R-200, Buchi). Finally, the concentrate was weighed and re-dissolved in 2 mL of methanol and filtered through a 0.22-μm filter. The concentration of the maize root exudate stock was 0.1 mg/mL and that of the collected fluids before concentration was 0.001 mg/mL. The root exudates were separated into two parts and prepared for antimicrobial assays and phenolic acid compound identification.

#### Antimicrobial Activity of Maize Root Exudates Against *P. sojae*

**FIGURE 2:**
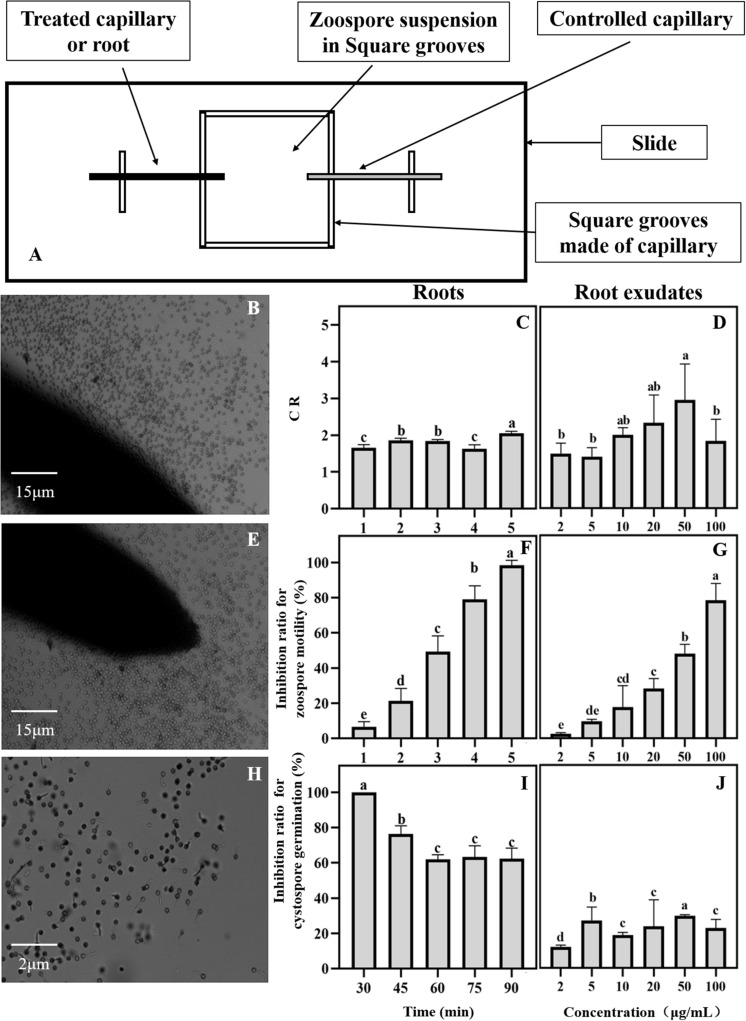
Effects of maize roots and exudates on zoospore behavior of *Phytophthora sojae.* **(A)** Chemotactic test device for maize root and exudates against *P. sojae* zoospores; **(B∼J)** Effect of maize roots and root exudates on zoospore chemotaxis roots: **(B,C)**; root exudates: **(D)**, swimming root: **(E,F)**; root exudates: **(G)**, and cystospore germination root: **(H,I)**; root exudates: **(J)**. Significant differences are based on ANOVA test (*P* < 0.05). The error bars indicate the standard errors of the means (*n* = 3).

The collection of maize root exudates for zoospores was performed according to Yang et al. (2014). The maize root exudate stock was diluted 2, 5, 10, 20, 30, and 50 times, and distilled water containing the same concentration of methanol was used as a control treatment. The chemotaxis of *P. sojae* zoospores toward maize root exudates was observed according to the method reported by Fan et al. (2002) with a few modifications. The specific method is shown in Figure 2. A square groove was made with a capillary measuring 1 mm in diameter, and then the square groove was placed on a glass slide (length 25 mm × width 25 mm × height 1 mm). A zoospore suspension at a concentration of 1 × 10^6^ cells/mL was added to the groove. One end was treated with a capillary tube to which a diluted root exudate was added. A capillary containing the same concentration of methanol at one end was used as a control, and the zoospore behavior was observed under a microscope.

The antimicrobial activity of maize root exudates against zoospore motility and cystospore germination was tested as described by Yang et al. (2014). In brief, 10 μL of root exudate and 40 μL of a zoospore suspension (10^6^/mL^–1^) or cystospore suspension (10^6^/mL^–1^) was immediately mixed on the glass slides. Then, the final concentration of the root exudates was diluted 5, 10, 25, 100, 150, and 250 times for concentrations of 100, 50, 20, 10, 5, 3.5, and 2 μg/mL on the slides. The slides were placed in Petri dishes containing moist filter paper and incubated at 25°C in the dark. Photographs of immobilized zoospores and germinated cystospores were taken under a microscope. The percentage of zoospores that encysted into cystospores was then recorded every 1 min for a period of 5 min, and the percentage of germinated cystospores was calculated after 1.5 h of incubation. Each treatment had three replicates.

### Identification of Phenolic Acid Compounds in Maize Root Exudates and Rhizosphere Soil

#### Gas Chromatography-Mass Spectrometry (GC-MS) Analyses of Root Exudates

The root exudates were derived by methoxypyridine and N-methyl-N-(trimethylsilyl)trifluoroacetamide. Then, the gas chromatography-mass spectrometry (GC-MS) fingerprints of the root exudates were obtained on a SHIMADZU GCMS-QP2010 instrument (SHIMADZU, Japan). The root exudates were separated on an SH-Rxi-5Sil MS capillary column (221-75954-30, 30 m × 0.25 mm × 0.25 μm, SHIMADZU). The pressure was maintained at 49.5 kPa, giving a column flow of 1 mL/min. The injection volume was 1 μL in splitless mode, and the injector temperature was 250°C. The initial column temperature was 40°C (hold 2 min), and it was increased at a rate of 3°C/min to 80°C and then increased to 260°C at a rate of 5°C/min, at which it was then held for 30 min. The ion source temperature was 230°C with an interface temperature of 250°C. Helium (99.999% purity) was used as the carrier gas at a flow rate of 1 mL/min. Mass spectra were obtained in electron impact (EI) ionization mode at 70 eV by monitoring the full-scan range (m/z 50-500). The compounds were identified by matching the mass spectra obtained with those of the reference compounds stored in the NIST14 library except for the compounds that appeared in the control. The characteristic fragments of the root exudate phenolic acids with more than 80% similarity were compared with those of the phenolic acid standards.

#### High-Performance Liquid Chromatography-Mass Spectrometry (HPLC–MS) Analysis of Rhizosphere Soil

The collection of rhizosphere soil was based on the methods of Bai et al. (2015) with slight modifications. In brief, maize or soybean plants were manually harvested from the pots, and large soil aggregates were removed by shaking the roots. The roots of all 15 plants derived from a single pot were pooled into a 50 ml EP tube containing 20 mL of sterile Silwet L-77 amended PBS buffer (PBS-S; 130 mM NaCl, 7 mM Na_2_HPO_4_, 3 mM NaH_2_PO_4_, pH 7.0, and 0.02% Silwet L-77) and washed on a shaking platform for 20 min at 180 rpm. The washing buffer was subjected to centrifugation (1,500 × g, 20 min), and the resulting pellet was defined as rhizosphere soil and then air dried. For the quantitative analysis of phenolic acids, 20 g of soil was extracted with 50 mL of 1 N NaOH and shaken for 12 h at room temperature (Kong et al., 2008). The filtrate was adjusted to pH 2.5 using HCl followed by centrifugation at 1200 *g* for 20 min. The phenolics were extracted from the acidified solution with ethyl acetate and re-dissolved in MeOH before being analyzed by HPLC-MS (Souto et al., 2000). In addition, phenolic acid analysis was performed in unplanted soil and was used as a control.

Based on the GC-MS results, five phenolic acids were identified from the root exudates and further selected to determine their presence and concentrations in maize and soybean rhizosphere soils by HPLC-MS. Cinnamic acid (Shanghai Yuanye Biotechnology Co., Ltd.), *p*-coumaric acid (Shanghai Yuanye Biotechnology Co., Ltd.), vanillic acid (Sigma-Aldrich Shanghai Trading Co., Ltd.), *p*-hydroxybenzoic acid (Shanghai Aladdin Biochemical Technology Co., Ltd.) and ferulic acid (Beijing Suo Laibao Technology Co., Ltd.) were purchased. The rhizosphere soil was analyzed using a Waters UPLC-MS system fitted with an Acquity UPLC System and a triple quadrupole mass spectrometer. Separation was performed on an Acquity UPLC BEH C18 column (1.7 μm, 2.1 mm × 50 mm). The solvents were as follows: solvent A, 0.1% glacial acetic acid (Aladdin Biochemical Technology, LC/MS grade) in water (Fisher Scientific, Shanghai, LC/MS grade) and solvent B, acetonitrile (Merck, HPLC grade). A multistep gradient was used for all the separations with an initial injection volume of 5 μL and a flow rate of 0.4 mL/min. The multistep gradient was as follows: 0–4 min 10–38% (v/v) solution B, 4.1–4.5 min 38–90% (v/v) solution B, 4.6–5.5 min 90% (v/v) solution B, and 5.6–8 min 90–10% (v/v) solution B. The column temperature was maintained at 40°C. The total run time was 8 min. The mass spectral ionization, fragmentation, and acquisition parameters were optimized on a tandem quadrupole mass spectrometer using electrospray ionization (ESI) in negative mode (Table 1). Quantification was performed in multiple reaction monitoring (MRM) mode with dwell and interscan delay times of 0.2 and 0.1 s, respectively. Data were acquired and processed using Masslynx software (version 4.0, Waters, Milford, MA, United States). The relative concentrations of phenolic compounds in the maize rhizosphere soil were calculated from the standard curves, which were generated from the areas of the different standard concentrations by HPLC-MS.

**TABLE 1:** Phenolic acids quantified by UPLC-MS in rhizosphere soil around maize and soybean.

Analytes	RT (min)	Transition	CV (v)	CE (v)	Molecular weight	Actual concentration (μg/g)		
						Maize	Soybean	Control
*p*-Hydroxybenzoic acid	0.85	136.968>93.476	28	32	137	17.11 ± 0.78a	3.29 ± 1.43b	0.82 ± 0.38c
*p*-Coumaric acid	1.34	162.968>119.468	28	32	164	26.13 ± 0.12	–	–
Cinnamic acid	2.80	147.032>76.967	26	30	148	0.15 ± 0.02	–	–
Vanillic acid	0.97	166.968>151.995	14	30	168	4.93 ± 0.13a	1.55 ± 0.10b	0.11 ± 0.02c
Ferulic acid	1.56	193.032>133.979	16	30	194	0.51 ± 0.00b	2.16 ± 0.06a	0.04 ± 0.02c

*RT, retention time; CV, cone voltage; CE, collision energy. Control, soil without plant culture. The error indicates the standard errors of the means (*n* = 3). Significant differences are based on ANOVA tests. Lower case letters show significant differences in the same phenolic acids at the soil with different plant culture at the 0.05 level.*

#### Antimicrobial Activity of Phenolic Acids Against *P. sojae*

The antimicrobial effect of phenolic acids from the maize exudates on the infection behavior of *P. sojae* (chemotaxis, zoospore motility, cystospore germination, and hyphal growth) was determined at different concentrations (cinnamic acid: 0.5, 1, 5, 10, and 20 mg/L, *p*-Coumaric acid: 1, 5, 10, 15, and 20 mg/L, and other phenolic acids: 1, 10, 20, 50, and 100 mg/L) with the same method as that used on the root exudates. In addition, the effect of the mixture (maize rhizosphere: five compounds, soybean rhizosphere: three compounds) and each single compound on *P. sojae* were further tested at concentrations of 0.1, 0.5, 1, 5, and 10 times their actual concentrations in the rhizosphere based on the HPLC-MS findings.

The effect of phenolic acids on *P. sojae* hyphal growth was evaluated as described by Miller et al. (1983) with a few modifications. The specific method employed 60 mL of V8 liquid medium with a 100 mL flask and each flask contained 6 dishes of *P. sojae*; the samples were cultured for 36 h on a shaking table at 28°C and 140 rpm. Then, and 600 μL phenolic acid solutions of different concentrations were added to each flask containing dishes. A 600 μL methanol solution without phenolic acid was added as the control; each treatment was repeated 3 times. After continuous culturing for 12 h, the liquid medium was removed by filtration; the hyphae were then wrapped in filter paper, dried and weighed to calculate the inhibition rate of the hyphae. The calculation method was as follows: mycelium inhibition rate (%) = (mycelia weight of control 1- treated mycelia weight)/(mycelia weight of control 1- mycelia weight of control 2) × 100%. Control 1 was the mycelial weight after treatment with 600 μL of methanol solution without phenolic acid, and control 2 was the mycelial weight after culturing for 36 h on a shaking table at 28°C and 140 rpm.

### Data Analysis

The experiments were set up using a completely randomized design. The yields, disease incidence in the intercrop and monoculture were analyzed by Independent Sample *t*-test; CR, and inhibition ratio of root exudates and compounds at different concentration were analyzed by one-way analysis of variance (ANOVA). Analyses were performed with an SAS software package (SAS Institute 2011), and the mean values (*n* = 3) were compared using Duncan’s new multiple range test at the 5% level. Figures were drawn using GraphPad Prism 8.

## Results

### Maize Intercropping With Soybean Can Restrict the Spread of *Phytophthora* Blight

As shown in Figure 1, the aboveground biomass of the soybean showed no significant difference between the intercrop and the monoculture (Figure 1C). In addition, the disease incidence of soybean *Phytophthora* blight was significantly decreased in the maize/soybean intercropping systems compared with that of the monoculture. The *Phytophthora* blight barely spread across the maize rows, and the disease incidence in center rows was also decreased by 15% (Figure 1F).

### Maize Roots and Root Exudates Interfere With the Infection Behavior of Zoospores

The interaction between the maize roots and *P. sojae* zoospores was observed under a microscope. The zoospores can be attracted to the maize roots (Figure 2B). Based on the CR values, the zoospores showed positive chemotactic activity toward the maize roots (Figure 2C). After approaching the maize roots, the zoospores rapidly lost their swimming ability and transformed into cystospores (Figure 2E). After 5 min, all the zoospores stopped swimming (Figure 2F). The maize roots then effectively inhibited the germination of the cystospores by 62% compared with the capillary tube control at 90 min (Figures 2H,I). The maize root exudates also showed a strong ability to attract zoospores (Figure 2D), inhibit the motility of the zoospores (Figure 2G), and suppress the germination of the cystospores (Figure 2J) in a dose-dependent manner. When the concentration of root exudates reached 20 μg/mL, which was close to the concentration of the root exudates before concentration, the inhibition rates for zoospore motility and cystospore germination reached 28% and 24%, respectively (Figures 2G,J).

### Phenolic Acids in Root Exudates and Rhizosphere Soils of Maize and Soybean

**FIGURE 3:**
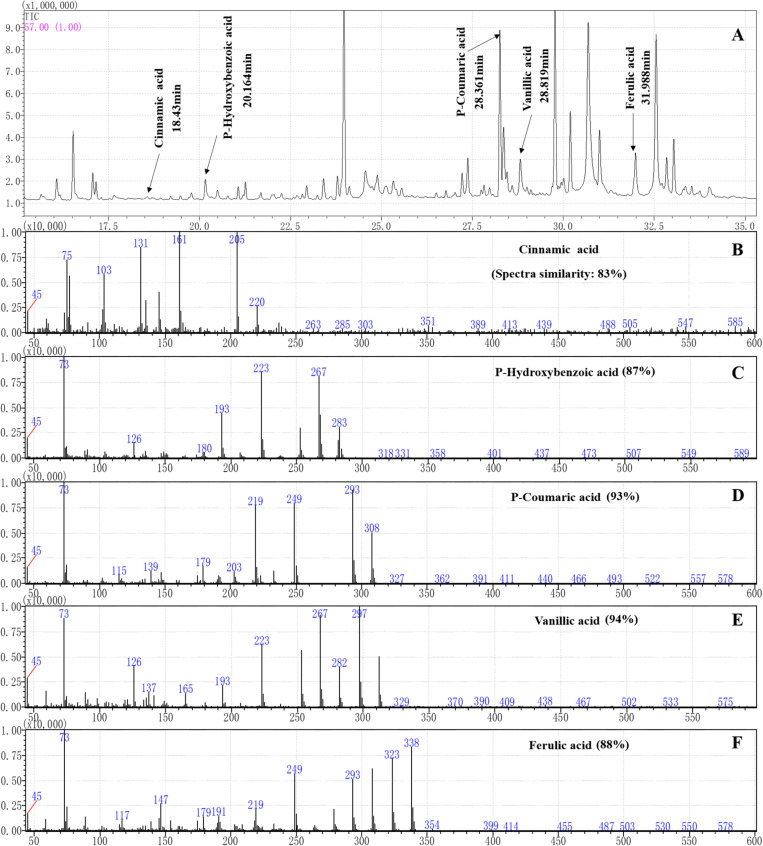
Separation and characterization of phenolic acids from maize root exudates by gas chromatography (GC) mass spectrometry (MS) analysis. **(A)** GC-MS profiles of root exudates showing five peaks at the following retention times (tr): 18.43 min (cinnamic acid), 20.164 min (*p*-hydroxybenzoic acid), 28.361 min (*p*-coumaric acid), 28.819 min (vanillic acid) and 31.988 min (ferulic acid) in the root exudates of maize were identified. **(B–F)** The characteristic ion fragment diagram and spectra similarities of the phenolic acids after derivation.

Sixteen organic acids, five alkanes, four alcohols, two amines and two esters were identified in the maize root exudates by GC-MS (Figure 3). Among them five phenolic acid compounds were consistently identified in the rhizosphere soil of maize with HPLC-MS (Table 1). The five phenolic acid compounds were *p*-coumaric acid, *p*-hydroxybenzoic acid, vanillic acid, ferulic acid and cinnamic acid in order of their concentration in the maize rhizosphere soil from high to low (Table 1). However, only three phenolic acid compounds, *p*-hydroxybenzoic acid, vanillic acid, and ferulic acid, were identified in the soybean rhizosphere soil by HPLC-MS, and *p*-hydroxybenzoic acid and vanillic acid showed significantly lower concentrations than those in the maize rhizosphere soil (Table 1). In addition, the *p*-hydroxybenzoic acid, vanillic acid and ferulic acid were also determined in the background soil, and the concentration was much lower than in the maize or soybean rhizosphere (Table 1), which illustrated that phenolic acids mostly measured in the soil was plant-derived.

### Phenolic Acid Compounds Show Various Activities Against *P. sojae* Infection

**FIGURE 4:**
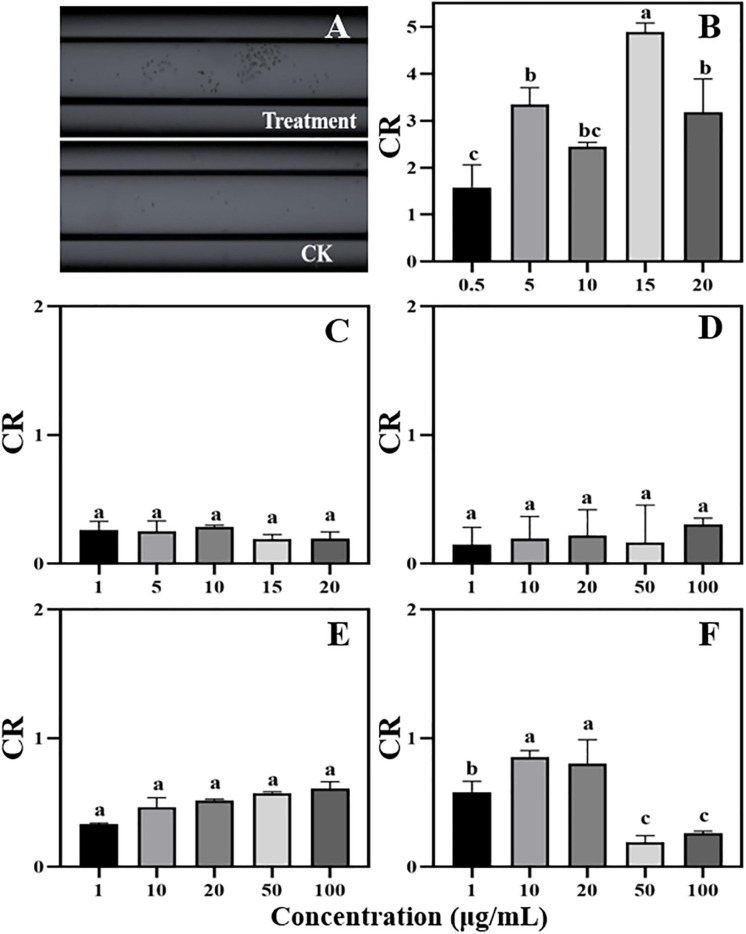
Chemotaxis of zoospores to different concentrations of phenolic acids. **(A)** Chemotaxis picture (treatment: 5 μg/mL cinnamic acid; CK: 1%MeOH-H_2_O); **(B)** Cinnamic acid (Concentration: 0.5–20 μg/mL); **(C)** *p*-Coumaric acid (Concentration: 1–20 μg/mL); **(D–F)** Vanillic acid, ferulic acid, and *p*-Hydroxybenzoic acid (Concentration: 1–100 μg/mL). Significant differences are based on ANOVA test (*P* < 0.05). The error bars indicate the standard errors of the means (*n* = 3).

**FIGURE 5:**
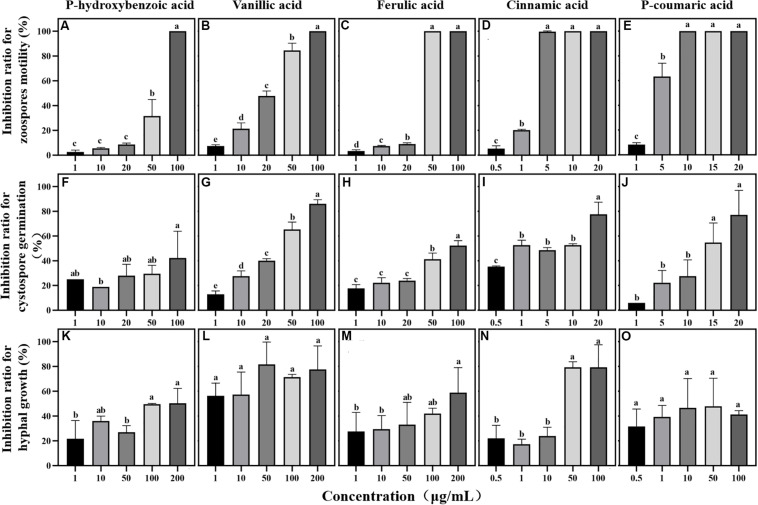
Effects of phenolic acids on different life stages of *Phytophthora sojae*. **(A–E)** Effects of five phenolic acids on zoospores motility; **(F–J)** Effects of five phenolic acids on cystospore germination; **(K–O)** Effects of five phenolic acids on hyphal growth. The concentration of phenolic acids was 0.5–100 μg/mL. The error bars indicate the standard errors of the means (*n* = 3). Significant differences are based on ANOVA tests. Lower case letters show significant differences in the inhibition effect at different stages at the 0.05 level.

Among the five phenolic acids, only cinnamic acid was associated with positive chemotaxis with *P. sojae* zoospores (Figure 4). However, the five phenolic acids showed dose-dependent inhibitory effects on the zoospore motility, cystospore germination, and hyphal growth of *P. sojae* (Figure 5). Cinnamic acid demonstrated the strongest activity against zoospore motility and cystospore germination, which were inhibited by 100% and 49%, respectively, at a concentration of 5 μg/mL (Figures 5D,I). The *p*-coumaric acid showed similar antimicrobial activity at 10–15 μg/mL (Figures 5E,J). Furthermore, vanillic acid and cinnamic acid showed the highest inhibitory activities against hyphal growth, with inhibition rates of 82% and 80%, respectively (Figures 5L,N). The remaining three phenolic acids also showed significant antimicrobial effects on zoospore motility, cystospore germination, and hyphal growth at soil concentrations of 10–50 μg/mL. In addition, when used at concentrations at which they are found in maize rhizosphere, phenolic acids such as *p*-coumaric acid and vanillic acid, could significantly interfere with the infection process of *P. sojae*, but they had little effect when used at those concentrations in the soybean rhizosphere (Tables 2, 3 and Supplementary Table S2). Then cinnamic acid could attract *P. sojae* zoospores at the rhizosphere concentration of 5 times and 10 times compared to the control (Supplementary Table S1). Interestingly, when these phenolic acid compounds were pooled together according to their concentrations in the maize rhizosphere soil, the mixture showed strong activity against all infection stages of *P. sojae*, except chemotaxis, in a dose-dependent manner (Table 2). The antimicrobial effect of the mixture on zoospore motility was significantly higher than that of phenolic acid alone. For example, the mixture (× 1) showed higher inhibitory activity against zoospore motility, which was 3.5 times that of the corresponding individual compounds at the same concentration (Table 2). However, the mixture of three phenolic acids at the soybean rhizosphere soil concentrations showed a slight effect on the infection processes, with the exception of cystospore germination. The inhibitory effect of the phenolic acid mixtures at the soybean rhizosphere soil concentrations and its proportions on cystospore germination were also significantly lower than they were at the maize rhizosphere soil concentrations (*t*-test, *p* < 0.05) (Table 3).

**TABLE 2:** The antimicrobial activity of phenolic acids against infection process of *Phytophthora sojae* according their concentrations in rhizosphere soil of maize.

Concentration	Compound	Chemotaxis	Inhibition ratio (%)		
			Zoospore motility	Cystospore germination	Hyphal growth
× 0.1 (0.1 times)	*p*-Coumaric acid	0.40 ± 0.12a	2.33 ± 1.20c	12.67 ± 0.33bc	37.33 ± 0.88a
	*p*-Hydroxybenzoic acid	0.53 ± 0.07a	4.67 ± 0.88b	8.33 ± 2.73c	9.00 ± 4.36b
	Vanillic acid	0.27 ± 0.07a	0.00 ± 0.00d	7.33 ± 2.73c	49.33 ± 6.36a
	Ferulic acid	0.20 ± 0.18a	0.00 ± 0.00d	7.33 ± 0.88c	37.00 ± 10.54a
	Cinnamic acid	0.13 ± 0.07a	4.00 ± 0.33bc	23.33 ± 4.05a	32.33 ± 13.20ab
	mixture	0.20 ± 0.12a	10.33 ± 0.88a	21.00 ± 4.00ab	32.33 ± 3.92ab
× 0.5 (0.5 times)	*p*-Coumaric acid	0.53 ± 0.07a	8.33 ± 1.76b	10.33 ± 3.18b	49.67 ± 1.45a
	*p*-Hydroxybenzoic acid	0.33 ± 0.07ab	8.33 ± 2.96b	10.00 ± 4.00b	23.33 ± 7.36b
	Vanillic acid	0.13 ± 0.07b	4.33 ± 1.45b	10.67 ± 2.19b	50.00 ± 3.61a
	Ferulic acid	0.47 ± 0.07a	4.67 ± 2.73b	8.67 ± 1.45b	25.33 ± 4.67b
	Cinnamic acid	0.33 ± 0.07ab	6.67 ± 2.33b	37.33 ± 7.84a	36.33 ± 9.87ab
	mixture	0.07 ± 0.07b	44.67 ± 0.88a	27.33 ± 11.67ab	43.67 ± 7.31ab
× 1 (1 times)	*p*-Coumaric acid	0.20 ± 0.07b	17.00 ± 4.73b	13.67 ± 2.73d	69.33 ± 15.41a
	*p*-Hydroxybenzoic acid	0.20 ± 0.12b	7.00 ± 2.08c	13.33 ± 2.03d	24.33 ± 8.09c
	Vanillic acid	0.07 ± 0.07b	6.67 ± 2.85c	23.67 ± 3.76c	33.67 ± 9.39bc
	Ferulic acid	0.07 ± 0.07b	6.67 ± 0.33c	27.67 ± 4.91bc	37.33 ± 4.91bc
	Cinnamic acid	0.97 ± 0.16a	15.67 ± 0.88b	37.33 ± 1.45b	25.67 ± 4.41bc
	mixture	0.90 ± 0.10a	60.00 ± 2.52a	50.00 ± 4.51a	52.67 ± 1.67ab
× 5 (5 times)	*p*-Coumaric acid	0.20 ± 0.07c	22.33 ± 1.76b	14.00 ± 1.53d	80.00 ± 2.52a
	*p*-Hydroxybenzoic acid	0.73 ± 0.24b	10.00 ± 2.31c	20.00 ± 2.89cd	29.67 ± 2.40c
	Vanillic acid	0.07 ± 0.07c	11.33 ± 1.20c	21.00 ± 3.46cd	40.33 ± 3.48c
	Ferulic acid	0.60 ± 0.07b	10.67 ± 0.88c	29.33 ± 3.28c	45.00 ± 16.52bc
	Cinnamic acid	1.27 ± 0.02a	18.67 ± 1.45b	43.67 ± 6.36b	37.00 ± 3.51c
	mixture	1.45 ± 0.02a	87.67 ± 0.67a	60.67 ± 2.40a	66.33 ± 7.88ab
× 10 (10 times)	*p*-Coumaric acid	0.40 ± 0.07c	29.33 ± 4.37c	57.33 ± 1.45b	90.67 ± 2.03a
	*p*-Hydroxybenzoic acid	0.53 ± 0.13c	21.33 ± 1.86cd	10.00 ± 1.00d	41.33 ± 7.31b
	Vanillic acid	0.13 ± 0.13c	19.67 ± 2.40d	18.67 ± 4.41c	66.00 ± 7.23a
	Ferulic acid	0.20 ± 0.13c	22.33 ± 3.18cd	56.33 ± 1.67b	31.00 ± 10.82b
	Cinnamic acid	1.30 ± 0.12b	38.00 ± 1.73b	58.00 ± 1.15b	41.00 ± 11.01b
	mixture	1.68 ± 0.13a	100.00 ± 0.00a	68.00 ± 1.73a	87.00 ± 2.52a

*Mixture is consisted of five phenolic acid compounds according to their concentrations and proportion in the rhizosphere soil of maize. The concentration with (× 0.1), (× 0.5), (× 1), (× 5) and (× 10) indicate that the concentrations of mixture were 0.1, 0.5, 1.0, 5.0 and 10.0 times of the rhizosphere soil concentration (Mixture = 0.15 mg/L cinnamic acid + 26.13 mg/L *p*-coumaric acid + 4.93 mg/L vanillic acid + 0.50 mg/L ferulic acid + 17.11 mg/L *p*-hydroxybenzoic acid). Different letters in the same group indicate significant differences by ANOVA (*P* < 0.05). The error indicates standard error of means (n = 3).*

**TABLE 3:** The antimicrobial activity of phenolic compounds against infection process of *Phytophthora sojae* according their concentrations in rhizosphere soil of soybean.

Compound	Inhibition ratio (%)			
	Chemotaxis	Zoospore motility	Cystospore germination	Hyphal growth
*p*-Hydroxybenzoic acid	0.25 ± 0.03a	13.00 ± 0.00a	2.33 ± 1.20c	−1.33 ± 2.85b
Vanillic acid	0.15 ± 0.02bc	5.00 ± 1.15b	18.00 ± 0.58b	17.67 ± 4.41a
Ferulic acid	0.11 ± 0.01c	10.00 ± 2.00a	11.33 ± 2.85b	25.00 ± 1.73a
mixture	0.18 ± 0.01b	3.67 ± 0.67b	27.00 ± 4.00a	21.33 ± 4.18a

*Mixture is the complex of three phenolic acids according to their concentrations and average proportion in the rhizosphere soil of soybean. The concentration indicates that the concentrations of mixture were 1.0 time of the rhizosphere soil concentration (Mixture = vanillic acid 1.55 mg/L + ferulic acid 2.16 mg/L + *p*-hydroxybenzoic acid 3.29 mg/L). Different letters in the same group indicate significant differences by ANOVA test (*P* < 0.05). The error indicates standard error of means (*n* = 3).*

## Discussion

### Non-host Plant Roots and Root Exudates Interfere With the Infection Behavior of *Phytophthora* Pathogens in Intercropping Systems

A large number of studies have found that maize intercropping with soybean, peppers, potatoes and other crops can not only reduce the occurrence of crop leaf diseases but also effectively inhibit the spread and expansion of soil-borne diseases (Sun et al., 2006; Li et al., 2009; Yang et al., 2014; Fu et al., 2016). In the present study, field experiments also indicated that maize intercropping with soybean could significantly inhibit the spread of *Phytophthora* blight in soybean in and across rows (Figure 1). Roots and root exudates play an important role in plant-microbe interactions in the rhizosphere (Bais et al., 2006; De-la-Peña et al., 2008; Doornbos et al., 2012). Our results indicated that maize roots and root exudates could attract *P. sojae* zoospores and suppress zoospore motility and cystospore germination, causing the pathogens to lose their infection ability (Figure 2). Therefore, the interference of maize roots and root exudates with the *P. sojae* infection process may be an important mechanism for inhibiting *Phytophthora* blight of soybean via maize/soybean intercropping. In addition, Yang et al. (2014) and Fang et al. (2016) found that maize and rapeseed roots could inhibit the growth of *Phytophthora in vitro* and decrease the incidence rate of *Phytophthora* blight in the field. Hence, the interference of non-host roots and root exudates on *Phytophthora* pathogens may play an important role in soilborne disease control via intercropped maize or rotated rapeseed.

### Phenolic Acids Show Various Abilities to Interfere With *P. sojae* Infection

Phenolic acids are complex and important secondary metabolites that are widely distributed throughout plant root exudates (Herrmann, 1989; Ryan et al., 1999). In the present study, five phenolic acid compounds (*p*-coumaric acid, *p*-hydroxybenzoic acid, vanillic acid, ferulic acid, and cinnamic acid) were consistently found in the root exudates and rhizosphere soils (Figure 3). Phenolic acids are important chemical substances in the rhizosphere of plants, and they can act as a signal to attract harmful and beneficial microorganisms within the soil (Liang et al., 2009; Kakkar and Bais, 2014). The zoospores of *P. sojae* displayed positive chemotaxis towards cinnamic acid but showed no response to the other four phenolic acids (Figure 4). Cinnamic acid could also serve as a signal substance for attracting *R. solanacearum* and accelerating disease progression in tobacco (Li B. et al., 2016). Previous research reported that cinnamic acid was found in the root exudates of a large number of plant species, including tobacco, cucumber, watermelon and rice (Ye et al., 2004; Hao et al., 2010; Ling et al., 2010; Li B. et al., 2016). Hence, cinnamic acid may be attracting *Phytophthora* zoospores to the roots of non-host plants. In addition, the previous research found the chemotaxis of beneficial microorganisms to cinnamic acid (Kape et al., 1991), indicating that non-host roots can also attract beneficial microorganisms to resist pathogens via cinnamic acid, which still requires further study.

Phenolic acids not only attract microorganisms as signal substances in soil but also directly affect the growth and reproduction of microorganisms (Yuan et al., 2018). In the present study, five phenolic acids inhibited the *Phytophthora* infection process in a dose-dependent manner (Figure 5). Among these compounds, cinnamic acid and *p*-coumaric acid, which are hydroxycinnamic acids, more strongly inhibited *P. sojae* zoospore motility and germination than the other phenolic acids (Figure 5). A large number of previous studies also reported the high antimicrobial activity of cinnamic acid and *p*-coumaric acid against plant pathogens (Zhang et al., 2007; Hao et al., 2010; Kim et al., 2012). Additionally, *p*-hydroxybenzoic acid has been reported to improve the growth of pathogens at low concentrations and creates a continuous soybean cropping barrier (Zhang et al., 2007). Previous studies also reported that some fungi could metabolize phenolic compounds and use them as carbon sources to obtain nutrition or to detoxify (Shalaby et al., 2012; Jin et al., 2020). Thus, we infer that the difference in the chemical structure may be an important reason for the different effects of phenolic acids on pathogens. Moreover, vanillic acid showed the highest inhibition ratio against *P. sojae* hyphal growth (Figure 5), indicating the different antimicrobial functions of phenolic acids in maize root exudates. This phenomenon has also been found in the interaction between plant pathogens and other antimicrobial compounds such as flavones, benzoxazinoids and antioxidants (Bagga and Straney, 2000; Lee and Bostock, 2007; Wang et al., 2020).

Apart from phenolic acids, organic acids, alkanes, alcohols, amines and esters were identified in the maize root exudates by GC-MS at the present study, which was also reported to have antimicrobial activity against pathogens, such as 3-Methylbutanoic acid in organic acid, tyrosol and D-pinitol in alcohols, and ethanolamine in amines (Lv et al., 2007; Wang et al., 2012; Abdel-Rhman and Rizk, 2016; Liu et al., 2019). Therefore these compounds may also participate in the suppression of *P. sojae* infection process by maize roots, but their involvement in this phenomenon needs further study. The antimicrobial substances in root exudates not only affect plant pathogens, but they also alter the soil microbial community around the roots (Hu et al., 2018; Stringlis et al., 2018; Huang et al., 2019). Previous studies have shown that soil microorganisms can affect pathogen infection process through microbiota-modulated immunity [MMI] and direct microbial competition [DMC] (competition for nutrients and space as well as the secretion of antimicrobials) (Hacquard et al., 2017; Vannier et al., 2019). Hence, root-secreted natural products, such as the various weapons in the chemical arsenal of plants, can help to resist pathogens.

### The Type and Concentration of Phenolic Acids in Host and Non-host Plants Mediate the Suppression of *P. sojae* by Intercropping

Whether phenolic acids inhibit or promote the growth of plant pathogens remains unclear (Ye et al., 2004; Zhang et al., 2006). On the one hand, phenolic acids such as cinnamic acid or *p*-coumaric acid (hydroxycinnamic acid) have been reported to have strong antimicrobial activity against crop pathogens (Kim et al., 2004; Hao et al., 2010); on the other hand, *p*-hydroxybenzoic acids, including vanillic acid (hydroxybenzoic acid), reportedly promote pathogen growth and cause soil sickness (Zhou and Wu, 2012a, b; Zhou et al., 2012). Therefore, the difference in the type of phenolic acids may be a factor in the inconsistency of their antimicrobial effects. In the present study, cinnamic acid and *p*-coumaric acid, which are types of hydroxycinnamic acid that have high inhibitory activity against *P. sojae*, were identified in maize rhizosphere soil but not in soybean rhizosphere soil (Table 1). Many previous studies also reported the absence of these hydroxycinnamic acids in the soybean rhizosphere (Zhang et al., 2007; Gao et al., 2014). Hao et al. (2010) found that the *p*-coumaric acid in rice root exudates could significantly inhibit the growth of *Fusarium oxysporum*, but it was not present in watermelon root exudates. These results indicated that non-host plant roots could secrete special phenolic acids (that are absent in the hosts) to suppress the soil-borne pathogen of the host plant, which may be an important mechanism underlying disease control by intercropping.

In addition, *p*-hydroxybenzoic acid and vanillic acid were also found in the soybean rhizosphere but much lower concentrations than in the maize rhizosphere (Table 1). The infection process was significantly disrupted by these phenolic acids at the maize rhizosphere concentrations but showed little effect at the soybean rhizosphere concentrations (Figure 5). *p*-Hydroxybenzoic acid and vanillic acid, which are hydroxybenzoic acids, were found in the root exudates of soybean and exhibited plant pathogenic fungi promotion at low concentrations and inhibition at high concentrations, for fungi such as *Aspergillus*, *Fusarium*, etc. (Zhang et al., 2007; Vio-Michaelis et al., 2012; Heleno et al., 2013). Therefore, the simultaneous presence of phenolic acids in non-host and host root exudates may increase their concentrations in the rhizosphere soil and result in a strong inhibition of *P. sojae* growth.

Synergism via the combination of two or more drugs has been utilized, and this combination/synergistic approach has demonstrated greater antimicrobial ability than single drugs alone (Sun and Johnson, 1960; Hossain et al., 2016). In the present study, the mixture of phenolic acids in maize rhizosphere soil showed higher inhibition during certain infection stages, especially zoospore motility, than the same concentrations of the individual compounds (Table 2). These results suggested the occurrence of synergistic antimicrobial effects, which have been widely used in pesticides for plant disease control (Chung et al., 2011; Berditsch et al., 2015). For example, a combined formulation of oregano and thyme showed a synergistic effect, resulting in enhanced efficiency against *Aspergillus. flavus*, *Aspergillus. parasiticus* and *Penicillium. chrysogenum* (Hossain et al., 2016). Hence, the synergistic interaction could improve the antimicrobial effect of phenolic acids on the infection behavior of *P. sojae.* In addition, we barely observed a synergistic antimicrobial effect with the mixture of phenolic acids at their soybean rhizosphere soil concentrations and proportions, which further supports this theory.

## Conclusion

We used maize and soybean intercropping systems as a model to determine whether non-host plant roots and root exudates can attract the zoospores of *Phytophthora* pathogens and then inhibit their growth and ability to cause infections, ultimately reducing the incidence of *Phytophthora* blight in the field. The difference in the type and concentration of phenolic acids between host and non-host plants was an important factor in the interference of non-host plant roots with *P. sojae* infection. Moreover, phenolic acids in maize root exudates exhibit synergistic antimicrobial activity, interfering with the infection behavior of *P. sojae* in soybean but not in soybean rhizosphere soil. This “non-host specificity” strategy can be used in agricultural systems to achieve sustainable and ecological disease management.

## Data Availability Statement

All datasets generated for this study are included in the article/Supplementary Material.

## Author Contributions

SZ and YXL conceived the ideas and designed the methodology. HZ, YY, YL, and JW performed the field experiment. HZ and YY performed GC-MS and HPLC-MS experiments. HZ, YY, YWL, and HW performed the biological activity test of standards. XM, HH, MY, and XH collected the data. HZ and YY analyzed the data. HZ, YY, SZ, and YXL wrote the manuscript. All authors contributed to the article and approved the submitted version.

## Conflict of Interest

The authors declare that the research was conducted in the absence of any commercial or financial relationships that could be construed as a potential conflict of interest.
