# Supplementary material for: Phenolic Acids Released in Maize Rhizosphere During Maize-Soybean Intercropping Inhibit Phytophthora Blight of Soybean
Source: Front Plant Sci. 2020 Jul 28;11:886. doi: 10.3389/fpls.2020.00886 (PMC7399372; doi:10.3389/fpls.2020.00886)
Supplement: Supplementary file 1 [file Data_Sheet_1.PDF]

Table S1 Effects of maize rhizosphere phenolic acids on the infection process of *P. sojae*

Concentration	Compound	The number of attracted zoospore		The rate of zoospore motility		The rate of cystospore germination		Hyphal growth weight	
		treatment	control	treatment	control	treatment	control	treatment	control
×0.1 (0.1 times)	mixture	7.33±4.06*	38.00±3.61	89.67±0.88*	100.00±0.00	59.00±3.00*	75.00±4.00	0.0784±0.0034	0.0862±0.0217
	p-Coumaric acid	14.00±4.16*	36.00±3.00	97.67±1.20	100.00±0.00	27.33±0.88*	31.33±0.88	0.0740±0.0007	0.0862±0.0217
	p -Hydroxybenzoic acid	16.00±4.04	29.67±6.36	95.33±0.88*	100.00±0.00	28.67±0.88	31.33±0.88	0.107±0.0027*	0.0601±0.0015
	Vanillic acid	6.67±1.76*	25.00±4.04	100.00±0.00	100.00±0.00	29.00±0.58	31.33±0.88	0.0828±0.0038*	0.0601±0.0015
	Ferulic acid	7.00±6.51	28.33±4.98	100.00±0.00	100.00±0.00	29.00±0.58	31.33±0.88	0.0902±0.0064*	0.0601±0.0015
	Cinnamic acid	6.00±3.06*	44.67±2.91	95.67±0.33*	100.00±0.00	24.00±1.53*	31.33±0.88	0.0930±0.0080	0.0601±0.0015
×0.5 (0.5 times)	mixture	2.33±1.86	36.33±12.12	55.33±0.88*	100.00±0.00	44.67±1.67*	75.00±4.00	0.0684±0.0061	0.0862±0.0217
	p -Coumaric acid	16.67±2.60	33.33±7.26	83.00±1.53*	92.67±0.33	46.67±1.76*	52.00±0.58	0.0632±0.0013*	0.0862±0.0217
	p -Hydroxybenzoic acid	9.33±2.85*	28.67±5.04	82.67±2.33*	90.33±0.67	47.00±2.52	52.00±0.58	0.0985±0.0045*	0.0601±0.0015
	Vanillic acid	4.00±2.00*	28.33±2.19	86.67±0.88*	92.67±0.33	46.67±1.45*	52.00±0.58	0.0825±0.0022*	0.0601±0.0015
	Ferulic acid	12.67±3.53	26.00±4.93	86.33±2.33*	92.67±0.33	47.67±2.73	52.00±0.58	0.0974±0.0027*	0.0601±0.0015
	Cinnamic acid	16.33±3.18*	50.33±9.84	84.67±2.03*	92.67±0.33	32.33±4.33*	50.67±0.88	0.0907±0.0059*	0.0601±0.0015
×1 (1 times)	mixture	54.33±1.86	61.33±5.90	40.00±2.52*	100.00±0.00	39.33±3.17*	75.00±4.00	0.0607±0.0014*	0.0862±0.0217
	p -Coumaric acid	7.67±2.60*	39.33±1.20	77.00±4.16*	92.67±0.33	50.33±2.33	58.33±3.28	0.0463±0.0134*	0.0862±0.0217
	p -Hydroxybenzoic acid	7.00±4.04*	31.00±5.03	86.00±2.08*	92.67±0.33	50.67±2.33	58.33±3.28	0.0983±0.0049*	0.0601±0.0015
	Vanillic acid	4.00±4.00*	39.67±10.40	87.00±2.00	92.67±0.33	47.33±3.53	58.33±3.28	0.0923±0.0055*	0.0601±0.0015
	Ferulic acid	2.67±2.67*	26.67±4.98	86.67±0.33*	92.67±0.33	42.00±1.53*	58.33±3.28	0.0900±0.0031*	0.0601±0.0015
	Cinnamic acid	66.00±9.29	69.67±6.39	78.00±1.15	92.67±0.33	39.00±0.58*	62.00±1.00	0.0971±0.0027*	0.0601±0.0015
×5 (5 times)	mixture	56.00±6.02*	38.67±1.76	12.30±0.67*	100.00±0.00	29.33±1.20*	75.00±4.00	0.0488±0.0069*	0.0862±0.0217
	p -Coumaric acid	7.33±2.33*	37.67±2.19	62.33±0.88*	80.33±0.67	60.33±1.45*	70.33±2.91	0.037±0.0021*	0.0862±0.0217

	p -Hydroxybenzoic acid	17.33±1.20	28.00±6.56	72.33±1.20*	80.33±0.67	56.00±2.31*	70.33±2.91	0.0946±0.0014*	0.0601±0.0015
	Vanillic acid	1.67±1.67*	26.33±3.38	71.33±1.45*	80.33±0.67	55.33±0.33*	70.33±2.91	0.0883±0.0022*	0.0601±0.0015
	Ferulic acid	18.00±1.15*	30.33±1.45	71.67±1.20*	80.33±0.67	50.00±4.04*	70.33±2.91	0.0856±0.0099	0.0601±0.0015
	Cinnamic acid	59.67±0.33*	47.00±0.58	65.33±0.67*	80.33±0.67	37.67±3.84*	67.00±2.00	0.0904±0.0021	0.0601±0.0015
×10 (10 times)	mixture	51.67±4.37*	31.33±4.10	0.00±0.00	100.00±0.00	24.00±0.58*	75.00±4.00	0.0308±0.0022*	0.0862±0.0217
	p -Coumaric acid	13.33±3.93	34.67±9.49	57.33±3.71*	81.00±1.53	33.67±1.67*	79.33±2.73	0.0280±0.0016*	0.0862±0.0217
	p -Hydroxybenzoic acid	15.33±4.67	28.00±3.06	63.67±0.33*	81.00±1.53	71.33±2.19	79.33±2.73	0.0876±0.0044*	0.0601±0.0015
	Vanillic acid	4.33±4.33*	30.33±3.18	65.00±1.52*	81.00±1.53	64.67±5.61	79.33±2.73	0.0728±0.0045*	0.0601±0.0015
	Ferulic acid	6.67±4.41	25.67±6.01	62.67±2.03*	81.00±1.53	34.67±2.33*	79.33±2.73	0.0938±0.0065*	0.0601±0.0015
	Cinnamic acid	59.00±3.51*	45.67±1.76	50.00±1.53	81.00±1.53	33.00±1.53*	79.33±2.73	0.0877±0.0067	0.0601±0.0015

Note: * indicates that the significant difference between the treatment and the control by t-test ($P<0.05$). The error indicates standard error of means (n=3).

Table S2 Effects of soybean rhizosphere phenolic acids on the infection process of *P. sojae*

Concentration	Compound	The number of attracted zoospore		The rate of zoospore motility		The rate of cystospore germination		Hyphal growth weight	
		treatment	control	treatment	control	treatment	control	treatment	control
×1 (1 times)	mixture	1.33±0.33	7.33±1.86	93.67±0.88*	97.33±0.33	35±2.00*	48±0.58	0.0624±0.0016*	0.0406±0.0071
	P-Hydroxybenzoic acid	3.00±0.58*	12.00±1.15	84.33±0.33*	97.33±0.33	54.67±3.71	56±4.00	0.0716±0.0012*	0.0406±0.0071
	Vanillic acid	1.67±0.33*	11.33±1.45	92.33±0.88*	97.33±0.33	42.33±0.33*	52±0.58	0.069±0.0018*	0.0406±0.0071
	Ferulic acid	1.00±0.58*	9.67±1.20	87.33±1.67*	97.33±0.33	49.67±4.41	56±4.00	0.061±0.0007*	0.0406±0.0071

Note: * indicates that the significant difference between the treatment and the control by t-test ($P<0.05$). The error indicates standard error of means (n=3).
